# Targeting the Siglec/sialic-acid axis in glioblastoma: Glycome-mediated immune evasion and emerging therapeutic platforms

**DOI:** 10.1093/noajnl/vdag234

**Published:** 2026-09-08

**Authors:** David Lee, William ElNemer, Omar Selim, Parth Patel, Sara Freeman, Hasan Slika, FNU Ruchika, Henry Brem, Christopher Jackson, Betty M Tyler

**Affiliations:** Department of Neurosurgery, Johns Hopkins University, School of Medicine, Baltimore, Maryland; Department of Neurosurgery, Johns Hopkins University, School of Medicine, Baltimore, Maryland; Department of Neurosurgery, Johns Hopkins University, School of Medicine, Baltimore, Maryland; Department of Neurosurgery, Johns Hopkins University, School of Medicine, Baltimore, Maryland; Baroda Medical College, Maharaja Sayajirao University of Baroda, Baroda, India; Department of Neurosurgery, Johns Hopkins University, School of Medicine, Baltimore, Maryland; University of Maryland, College Park, Maryland; Department of Neurosurgery, Johns Hopkins University, School of Medicine, Baltimore, Maryland; Department of Neurosurgery, Johns Hopkins University, School of Medicine, Baltimore, Maryland; Department of Neurosurgery, Johns Hopkins University, School of Medicine, Baltimore, Maryland; Department of Neurosurgery, Johns Hopkins University, School of Medicine, Baltimore, Maryland; Department of Neurosurgery, Johns Hopkins University, School of Medicine, Baltimore, Maryland

**Keywords:** glioblastoma, immune-checkpoint blockade, immunotherapy, sialic acid, Siglec

## Abstract

Despite encouraging advances in cancer immunotherapy, several malignancies leverage resistance pathways to escape the effects of treatment. Abnormal cell signaling and cytokine secretion modulated by cancer cells promote a tumor microenvironment (TME), wherein the efficacy of infiltrating immune cells is diminished. There is increasing evidence that alteration of the glycosylation profile of some TMEs is a key factor in exhausting the immune response. The sialic-acid/Siglec axis has received attention for its suspected involvement in this context through both preclinical study and several clinical trials. Against this backdrop, glioblastoma (GBM) is a well-established immune-suppressive tumor with disappointing responses to most traditional immunotherapies. Recent studies have suggested that GBM can exploit the sialic-acid/Siglec axis to promote immune evasion and resistance to traditional immune checkpoint inhibitors. This review examines all presently identified Siglec interactions and the current state of Siglec-centered therapies with respect to GBM.

Key Points

- Siglec and sialic-acid interactions can mediate tumoral immunosuppression.
- Glioblastoma exhibits a unique, hypersialylated glycome.
- Siglec/sialic-acid disruption may enhance glioblastoma immunotherapy

Glioblastoma (GBM) is one of the most aggressive and treatment-refractory malignancies, demonstrating resistance to most clinically implemented immunotherapies.^1^ Three phase III clinical trials investigating immune checkpoint inhibitor efficacy in GBM involving inhibition of programmed death-1 (PD)-1/programmed death ligand-1 (PD-L1) interactions (nivolumab) have failed to improve overall survival.^2-4^ A key obstacle to the ability of immunotherapies to achieve a clinically significant improvement in survival is GBM’s highly immunosuppressive ­microenvironment. The immune profile of GBM’s tumor microenvironment (TME) is predominated by myeloid populations such as tumor-associated macrophages and microglia (TAMs) and myeloid-derived suppressor cells (MDSCs), which generally promote tumor growth by inhibiting cytotoxic T cell activity.^5^

One mechanism implicated in GBM’s immunosuppressive TME is alteration of glycosylation, particularly the abnormal upregulation of sialic acid residues, or hypersialylation, of tumor-cell surfaces.^6^^,^^7^ In fact, unique genetic profiles modulating glycosylation in gliomas have been discovered.^8^ GBM displays a glycosylation profile skewed toward α2,3 and α2,6 sialylated N-glycans,^9^^,^^10^ a phenotype driven by dysregulation in various sialyltransferases (eg *ST6GAL1*, *ST3GAL* family)^9^^,^^11^^,^^12^ and sialidases.^13^ These alterations can obscure antigens on tumor cell surfaces that would leave cancers vulnerable to immune cell recognition, serving as an immune escape pathway for various solid tumors.^7^

Sialic acid groups are known to exert most of these effects through their interactions with Siglecs, or sialic-acid binding Ig-type lectins, which are a family of cell-surface receptors expressed in a variety of immune cell types.^14^ In normal physiology, Siglecs are suspected to attenuate autoimmune responses or excessive immune activation. However, various models of cancer have demonstrated exploitation of Siglec recognition to inhibit immune-mediated oncolysis and facilitate immunotherapy resistance.^15^ Transcriptomics of human glioma samples have reported increased Siglec-positive tumor-infiltrating immune cells and strong associations between aberrant Siglec expression and poorer patient outcomes, including diminished survival, higher tumor grade, and upregulation of tumorigenic TAM phenotypes.^16-18^

The plethora of oncogenic interactions identified in the Siglec/sialic-acid axis and their relative novelty within the GBM literature warrant review for their implications in Siglec-directed GBM immunotherapy. Here, we review currently known Siglec and sialic acid interactions in preclinical and translational studies of GBM, aiming to distinguish the mechanisms by which each contributes to immune resistance and evaluate the therapeutic strategies proposed to target these mechanisms.

## Molecular Landscape of GBM Sialylation

GBM expression of mannose-, fucosylated-, and biantennary-bisecting N-glycans are distinctly altered when compared to healthy brain tissue and other ­malignancies.^9^^,^^10^^,^^13^ N-glycan MALDI-MSI of GBM tissue revealed increased intensity of α2,3 sialylated biantennary N-glycan signal species, including those at mass/charge ratio of (m/z) 2,137 (Hex5dHex1HexNAc4 + 1Sia2,3) and α2,6 sialylated trian­tennary N-glycans at m/z 2,127 (Hex5dHex1HexNAc4 + 1Sia2,6) compared to NBT and oligodendroglioma (OGD), which predicted worse prognosis.^9^ Broader shifts toward α2,3 glycosidic linkages of sialic acid N-acetylneuraminic acid (NeuAc) have also been associated with oncogenic transformation and tumor initiation of glioma stem cells (GSC).^9^^,^^19^ Analysis of high-grade glioma cell populations identified a substantial elevation of Sialyl Lewis X (SLe^x^), terminal glycans that are α2,3-sialylated and α1,3 fucosylated; presence of SLe^x^ is potentiated by increased *MGAT5*-mediated synthesis of multi-antennary N-glycans with β1,6-GlcNAc branching.^10^ Other sialyl molecules associated with GBM include polysialic acid (polySia), representing α2,8-linked sialic acid chains that primarily attach to neural cell adhesion molecule (NCAM) and potentially drive GBM invasion.^20^

The enzymes that drive these changes include dysregulated sialyltransferases and sialidases.^13^^,^^21^  *ST6GAL1*, which catalyzes α2,6 linked sialylation, is abnormally upregulated in GBM progression and is implicated in the maintenance of a brain tumor-initiating cell (BTIC) phenotype.^9^^,^^11^^,^^12^ By contrast, *ST6GAL2* expression is decreased in GBM compared to NTB; while, in this case, its mechanistic effects are uncharacterized, its lower expression is associated with worsened prognoses, suggesting a suppressive role in GBM.^9^^,^^12^ Other examples include members of the *ST3GAL* family (eg *ST3GAL3* and *ST3GAL4*), which are upregulated drivers of α2,3-linked sialylation.^13^ Members of the *ST8SIA* family, notably *ST8SIA2* and *ST8SIA4*, encode enzymes that transfer sialic acids via α2,8-linkages and appear uniquely responsible for the creation of polysialic acid chains (polySia) in GBM tumors.^20^^,^^21^ Further, dysregulation of enzymes that cleave terminal sialic acid sialidases, or neuraminidases, has been identified in GBM, albeit sparsely.^13^  *NEU3* has been reported to have reduced expression in human GBM tumors, suggesting an immunosuppressive role. On the other hand, increased expression of *NEU3* and *NEU4* in certain GBM subpopulations implicates heterogeneous sialidase activity and a rapid turnover of surface sialylated glycans, aligning with findings of high *de novo* sialylation rates in GBM cells.^13^ This complex dynamic of sialyltransferases and sialidases contributes to the overall highly dynamic sialylation and desialylation in GBM.^13^

The roles of aberrant α2,3/α2,6 glycosidic linkage and polySia expression profiles, as well as dysregulation of sialic-acid-modifying enzymes, have been implicated in a variety of oncogenic mechanisms, ranging from tumor initiation and intratumoral connectivity to metastasis and therapy resistance.^9-13^ The unique characterization of GBM’s sialylation profile, summarized in Figure 1, should offer insight into ongoing studies of Siglec-mediated interactions in GBM tumors, which may in turn provide mechanistic reasoning for described associations between sialylation and tumor aggressiveness.

**Figure 1.**
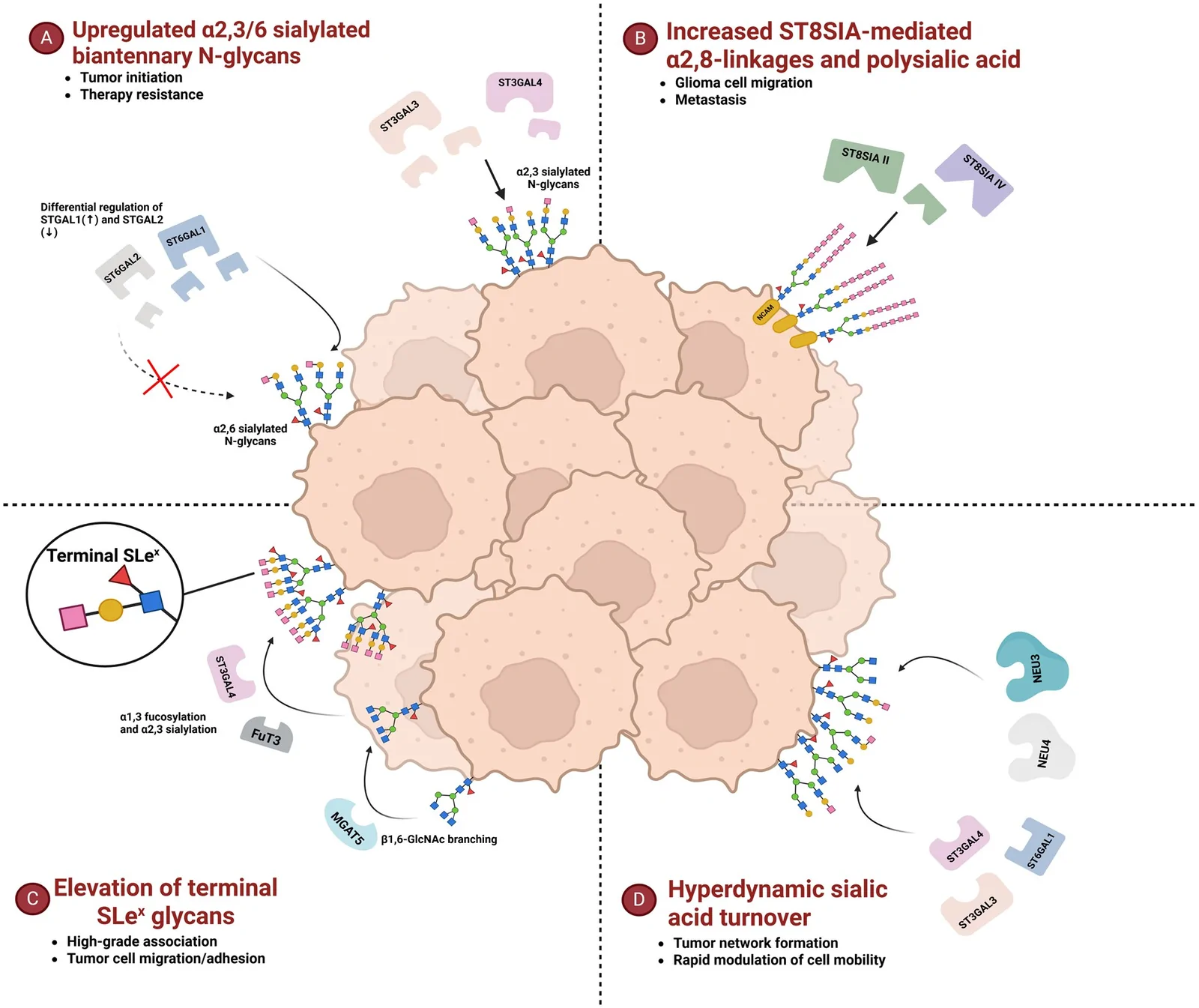
Differential expression of STGAL enzymes in GBM may contribute to abnormal sialylation in the form of α2,3/6 sialylated N-glycan residues **(A)**. A distinct ST8SIA-α2,8-linkage axis represents a potential, aberrant driver of the polysialic-acid-rich GBM microenvironment **(B)**. Specific high-grade glioma cell populations with aggressive migratory behavior are distinguished by an association with excess terminal SLe^x^ glycans **(C)**. GBM populations demonstrate both increased sialidase activity and increased *de novo* sialylation rates, suggesting that sialylation plays a highly dynamic and modulable role within the GBM glycome **(D)**. Created in BioRender. Slika, H. (2026) https://BioRender.com/tj43ul7. Abbreviations: ST6GAL, ST6 beta-galactoside alpha-2,6-sialyltransferase; ST3GAL, ST3 beta-galactoside alpha-2,3-sialyltransferase; ST8SIA, ST8 alpha-N-acetyl-neuraminide alpha-2,8-sialyltransferase; NCAM, neural cell adhesion molecule; SLe^x^, sialyl-lewis^x^; FuT, fucosyltransferase; MGAT, Mannosylglycoprotein N-acetylglucosaminyltransferase; GlcNAc, N-acetylglucosamine; NEU, neuraminidase.

## Siglec-Specific Interactions

### Siglec-3

Siglec-3 (CD33), as the eponymous member of the CD33-related Siglec family, is expressed on cells of myeloid lineage, with highest enrichment in early progenitor cells.^22^^,^^23^ Siglec-3 recognizes and binds α2,3- and α2,6-linked sialic acids, thereby activating immunoreceptor tyrosine-based inhibitory motifs (ITIMs) and recruiting SHP-1 and SHP-2 phosphatases. These phosphatases dephosphorylate motifs and kinases critical to immune activation pathways (eg nuclear factor-κB), including immunoreceptor tyrosine-based activation motifs (ITAMs) and spleen tyrosine kinase (Syk).^22^^,^^23^

High expression of Siglec-3 in undifferentiated myeloid cells and its immunosuppressive functions have motivated studies into its oncogenic effects, chiefly in acute myeloid leukemia.^24^^,^^25^ However, Siglec-3’s involvement in GBM remains relatively underexplored. One potential area of association involves Siglec-3’s constitutive expression on MDSC, which are notably elevated in GBM-patient blood samples compared to healthy controls and patients of other cancer types.^26-28^ Growing evidence reinforces the tumorigenic properties of certain MDSC populations, which are closely linked to T cell and dendritic cell (DC) suppression, potentiation of immunosuppressive natural killer (NK) cell phenotypes, and enhanced tumor growth.^28-30^ Interestingly, MDSCs have been shown to interact with pro-inflammatory S100A8/A9 signaling via Siglec-3-mediated recognition, thereby exerting counteractive, immune-suppressive effects in cancer.^27^ Such mechanisms reinforce observations in GBM models detecting restoration of T cell activity following anti-Siglec-3 targeting of MDSCs.^28^

Prior findings position Siglec-3 as a potential contributor to myeloid-facilitated immune suppression in GBM, particularly through MDSC activity. However, Siglec-3 itself is not specific for MDSCs and may play a broader, yet uncharacterized role in other immune cell populations. Future studies should identify its precise functions in GBM, whether through MDSC-dependent pathways or other myeloid lineage populations.

### Siglec-5

Siglec-5 (CD170) is a CD33-related receptor expressed on subsets of CD4^+^/8^+^ T cells, neutrophils, and monocytes.^31^^,^^32^ Like Siglec-3, Siglec-5 recruits SHP-1/2 phosphatases through ITIM activation to facilitate myeloid function but is also implicated in T cell suppression via inhibition of T-cell receptor (TCR)-induced transcription factors like NFAT and AP-1.^31^ Soluble Siglec-5 has been shown to interact with putative sialylated ligands on several tumor cell lines, interfering with activation of tumor-specific activated T cells, and positioning Siglec-5 as an effector of both myeloid and lymphoid populations.^31^

In GBM-patient-derived samples, expression analyses have detected enrichment of Siglec-5 alongside other CD33-related Siglecs, albeit within distinct immune subsets. Where other Siglecs are most abundant on myelocytic (M)-MDSCs, Siglec-5 is preferentially expressed on circulating and intratumoral polymorphonuclear (PMN)-MDSCs.^33^ Moreover, Siglec-5 ligands were sparsely expressed on glioma cells themselves; these ligands were instead also detected in PMN-MDSC subsets, suggesting yet uncharacterized mechanisms for inter-immune-cell reactivity within the GBM TME.^33^

Although dedicated studies of Siglec-5 in GBM are limited, its unique expression on PMN-MDSCs highlights potential for combined blockade strategies that target both M- and PMN-MDSC-mediated immunosuppression.

### Siglec-7/9

Siglec-7 (CD328) and Siglec-9 (CD329) are CD33-related Siglecs expressed on myeloid-derived cells, NK cells, and T cell subsets.^34^^,^^35^ The receptors recognize and bind to overlapping ɑ2,3 glycans synthesized by *ST3GAL1* and *ST3GAL4*.^36^ Like Siglec-5, they initiate ITIM-mediated immunosuppressive cascades and suppress T cell activation.^35^^,^^36^ Moreover, while both receptors are expressed on NK cells, Siglec-7 is uniquely found to inhibit NK-cell activation via its disruption of NK cell and target cell contact.^34-36^ Nevertheless, Siglec-7 and 9 are frequently identified as potential co-targets of interest in various cancers.^34^^,^^36^

Prior single-cell transcriptomic analyses of glioma cells have established correlation of increased Siglec-7 and Siglec-9 expression with higher tumor grade, mesenchymal subtype, and poorer survival.^37^ Molecularly, elevations in either receptor type are linked to increased infiltration of M2-macrophages and diminished T-cell-mediated cytotoxicity. Siglec-9 ablation has notably shown explicit improvements in T cell and macrophage-mediated GBM killing.^16^^,^^18^ These effects have implicated Siglec-7 and Siglec-9 in distinct synergistic relationships with other immune checkpoints. For example, macrophages and TAMs with elevated expression of either receptor have shown concomitant expression of TIM-3.^37^ Siglec-7 expression has also been linked with co-expression of PD-1, CTLA-4, and TIGIT among other immunosuppressive markers, and may play a role in the upregulation of immune checkpoints.^16^ On the other hand, Siglec-9+ TAM subsets accumulate and persist through anti-PD-1 therapy, indicating Siglec-9 as a potential facilitator of immune escape in GBM cells subjected to ­traditional immunotherapy.^18^

Siglec-7 and Siglec-9’s roles in T and NK cell suppression are complemented by their unique associations with a broad panel of immune checkpoints and persistence through PD-1 blockade, suggesting that both Siglecs may serve as effective targets in both individual and combination blockade therapies.

### Siglec-10

Siglec-10 is a CD33-related receptor broadly expressed on neutrophils, eosinophils, NK cells, monocytes, and B cells.^14^^,^^38^ It localizes to TAMs where macrophage-bound Siglec-10 interacts with heat-stable antigen or CD24 on tumor-cell surfaces and induces “don’t eat me” signaling. Siglec-10 thereby prevents phagocytosis and is positioned as a key TAM-associated immune checkpoint in cancer TMEs.^39^^,^^40^

Siglec-10 delivers inhibitory signals through its ITIM motifs, recruiting SHP-1 and SHP-2 phosphatases to dampen immune responses.^14^^,^^41^ It interacts with several ligands, with those most pertinent to cancer including CD24 and CD52. CD24, a sialylated, GPI-anchored receptor, is highly expressed in GBM and functions as an antiphagocytic signal. By engaging Siglec-10 on TAMs, CD24 suppresses Toll-like receptor (TLR)-mediated inflammation and inhibits phagocytosis, akin to the CD47–SIRPα and PD-1/PD-L1 pathways.^39^^,^^42^ Interaction of CD52 with Siglec-10 on T cells suppresses proliferation and activation via dephosphorylation of ZAP-70 and Lck.^41^^,^^43-45^ CD52 may also contribute to PMN-MDSC–mediated immunosuppression.^46^^,^^47^

Expression studies show Siglec-10 is enriched in mesenchymal GBM subtypes and high-grade gliomas, often correlating with poor prognosis. However, such findings remain inconsistent.^33^^,^^37^^,^^48^^,^^49^ Mao et al. associated Siglec-10 expression with immunosuppressive cytokines and cell populations, while Wang et al. linked it to proangiogenic and inflammatory pathways that disrupt the blood-brain barrier and recruit tumor-promoting immune cells. Both studies, however, utilized transcriptomic and functional enrichment analyses, highlighting the need for experimental validation.^48^^,^^49^

Siglec-10’s implicated role in driving immune-suppressive macrophage phenotypes through interactions with ligands such as CD24 and CD52 makes it an attractive target in a myeloid-dominated GBM TME. These promising findings have recently advanced an anti-Siglec-10 antibody (ONC-841) to clinical trials for advanced solid tumors. This advancement is motivated by growing preclinical evidence that CD24/Siglec-10-targeted therapy may be a potent expansion to antitumoral immune modulation.^39^^,^^51-58^ Notably, the Phase 2 portion of this trial includes a GBM-dedicated cohort that will assess the efficacy of ONC-841 combined with standard of care in patients with GBM.^50^

### Siglec-15

Siglec-15 is an evolutionarily conserved receptor expressed on myeloid cells, particularly peritumoral CD68+ TAMs.^59^^,^^60^ While the scope of its binding partners and the signaling pathways they induce are yet to be fully elucidated, studies have identified specific α2,6-linked-sialic acid residues to be likely binding candidates, including ligands such as Sialyl-Tn (sTn) and CD11b.^61-63^ For example, solid tumors overexpressing sTn will interact with Siglec-15+ TAMs, increasing the levels of TGF-β (an immune regulatory cytokine) produced by the interacting TAMs.^62^

Prior transcriptomic analyses indicate that Siglec-15 may function as a poor prognostic biomarker in glioma by modulating TAM activity and promoting tumor progression.^59^ Its expression has been significantly associated with *TMED2*, which is implicated in glioma cell growth, migration, and invasion.^64^ Moreover, Siglec-15 expression demonstrates an inverse correlation with PD-L1 in both GBM and lower-grade glioma (LGG) tissues, a finding validated by experimental studies.^60^ This suggests that even when the PD-1/PD-L1 axis is inhibited, Siglec-15 may continue to suppress antitumor immunity. Finally, Siglec-15 is presently one of few Siglecs directly modulated in GBM studies. Siglec-15 deletion in GL261 models demonstrated increased macrophage M1-polarization and subsequent antitumoral functions both in culture and in mice.^59^^,^^60^

Siglec-15 represents one of the best characterized ­members of the Siglec family in studies of cancer immunosuppression and is one of few that is presently targeted in clinical trials.^65^ Its unique expression on cell populations lacking in PD-1 provides evidence for its potential status as an immune-escape tool in GBM and suggests that future studies should consider leveraging immunotherapy-resistant models in their analyses.

### Siglec-11/16

Siglec-11 and Siglec-16 are paired receptors expressed on macrophages and microglia; despite high genetic homology and similar glycan binding preferences, they are implicated in opposing inhibitory and activating functions.^66^^,^^67^ Specifically, Siglec-11 receptors recognize and bind to polySia residues,^68^^,^^69^ thereby inducing ITIM activation, downregulating ITAM signaling, and suppressing pro-inflammatory and phagocytic responses.^68-70^ By contrast, Siglec-16 is distinguished by its association with DAP12, an ITAM-bearing molecule that can crosslink with partner membrane receptors to induce macrophage expansion and inflammatory signaling.^71^

Direct alteration of Siglec-11 or Siglec-16 and its impacts on GBM have been relatively underexplored. However, their roles in modulation of the neuroinflammatory response pose important implications for the GBM TME. The inhibitory effect of Siglec-11 activity induces macrophage M2 polarization and cancer progression in other cancer models, including gastric cancer.^72^ Prior detection of Siglec-11 mRNA upregulation in human GBM tumors expands the concern that GBM may also co-opt Siglec-11’s immune-suppressive activity.^73^ On the other hand, the presence of functional Siglec-16 alleles in GBM patients has been correlated with increased overall survival.^74^ This survival is further increased with the presence of polySia on tumor cells, indicating both Siglec-11 and Siglec-16 may interact with tumor-expressed polySia ligands.^74^

Despite relatively limited study of the Siglec-11/16 axis in GBM, the findings described illustrate its complex mechanism involving both immunogenic and immune-suppressive activities. Moreover, the similarities between both Siglecs in structure and implicated ligand-binding preferences pose a unique challenge for future therapeutic approaches, which will need to account for strong Siglec-11 and -16 discrimination to achieve optimal antitumoral effects.

All receptors and their associated findings described above are summarized in Table 1.

**Table 1.** Summary of Siglec receptor types that have been investigated in the context of cancer and/or glioblastoma*

Siglec Name	Immune cell expression profile	Immune mechanism	Associations with GBM	Effects of direct modulation on GBM (*In Vitro*)	Effects of direct modulation on GBM (*In Vivo*)	Other cancers
Siglec-3 (CD33)^22^ ^,^ ^23^ ^,^ ^28^ ^,^ ^30^	- Myeloid lineage cells - Decreasing enrichment with cell maturity	ITIM-mediated SHP-1/2 cascade	- Siglec-3-rich MDSCs elevated in GBM compared to other cancers and healthy controls	- Anti‑Siglec-3 depletion/of MDSCs from GBM patient samples restores T‑cell function in co‑culture.	N/A	AML
Siglec-5 (CD170)^31-33^	- Activated CD4+/CD8+ T cells - Myeloid lineage cells - Preferentially enriched on PMN-MDSCs	- ITIM-mediated SHP-1/2 cascade - Disrupts TCR-driven NFAT/AP-1 signaling	- Highly expressed on intratumoral PMN-MDSCs - GBM cells express few Siglec-5 ligands	N/A	N/A	N/A
Siglec-7 (CD328)^16^ ^,^ ^17^ ^,^ ^34-36^	- NK cells - Activated CD4+/CD8+ T cells - Myeloid lineage cells	- ITIM-mediated SHP-1/2 and ZAP-70 cascade - Disrupts TCR-driven NFAT/AP-1 signaling - Blocks NK-target cell contact and subsequent NK cell activation	- Associated with high tumor grade, mesenchymal subtype, and poorer survival - High expression linked to greater M2 TAM infiltration and diminished T-cell cytotoxicity - Coexpressed with PD-1, CTLA-4, and TIGIT immune checkpoints	N/A	N/A	Pancreatic; Prostate
Siglec-9 (CD329)^17^ ^,^ ^18^ ^,^ ^34-36^	- NK cells - Activated CD4+/CD8+ T cells - Myeloid lineage cells	- ITIM-mediated SHP-1/2 and ZAP-70 cascade - Disrupts TCR-driven NFAT/AP-1 signaling	- Associated with high tumor grade, mesenchymal subtype, and poorer survival - High expression linked to greater M2 TAM infiltration and diminished T-cell cytotoxicity - Siglec-9 TAMS accumulate following anti-PD-1 therapy	- Siglec-9 deletion in macrophages increased production of inflammatory chemokines (eg CCL4, CCL8, etc.) and T cell recruitment - Siglec-9-deficient macrophages increased IL4 and IL10 production	- Siglec‑9 ablation in murine GBM models reversed immunosuppressive macrophage activity, improving lymphocytic infiltration and tumoricidal function	Pancreatic; Prostate
Siglec-10^38-43^	- Broadly expressed on both myeloid and lymphoid lineage cells - Unique eosinophil expression	- ITIM-mediated SHP-1/2 cascade - CD24 binding suppresses TLR-mediated phagocytosis - CD52 binding dephosphorylates ZAP70 and Lck, suppressing T cell effector functions	- Enriched in mesenchymal and high‑grade gliomas - Associated with immunosuppressive cytokines, angiogenesis, BBB disruption, and poor prognosis	N/A	N/A	Breast; Hepatocellular carcinoma; Pancreatic; Mantle‑cell lymphoma; Chronic lymphocytic leukemia
Siglec-15^59-62^ ^,^ ^64^ ^,^ ^66^	- Myeloid lineage cells - Richly expressed on TAMS and some tumor cell types	- Unclear mechanism but likely binds to residues containing α2,6-linked sialic acids (e.g. sTn, CD11b) - Suspected to suppress macrophage activity via upregulation of TGF-β secretion	- Upregulated in gliomas but correlates inversely with PD-L1 expression - Associated with TMED2 expression, immunosuppressive TAM activity, and poor prognosis	- Siglec-15 KO upregulated M1-related chemokine expression and phagocytosis in macrophages - Greater T cell proliferation and activation in coculture with Siglec-15 KO macrophages	- Siglec‑15 ablation increases immune infiltration of tumors and synergizes with anti‑PD‑1 - Anti‑Siglec‑15 antibody therapy combined with ZIKV oncolytic virus induces tumor regression.	NSCLC; Colon
Siglec-11/16^66^ ^,^ ^68^ ^,^ ^69^ ^,^ ^71^ ^,^ ^72^ ^,^ ^74^	- Primarily macrophages and microglia	- Siglec-11 and polySia binding induces ITIM-mediated immunosuppression - Siglec-16 associates with DAP12 and activates ITAM signaling	- Siglec-11 mRNA is upregulated in GBM tumors - Siglec-16 allele improves survival in GBM patients; correlates with polySia expression	N/A	N/A	Gastric

* Siglecs not listed (eg Siglec-1, -2, -4, -6, etc.) were excluded due to sparsity of differential expression, functional, and/or prognostic data specifically in GBM.

Abbreviations: GBM, glioblastoma; ITIM, immunoreceptor tyrosine-based inhibitory motif; SHP, src homology 2 domain-containing protein tyrosine phosphatase; MDSC, myeloid-derived suppressor cell; AML, acute myeloid leukemia; PMN, polymorphonuclear; TCR, T-cell receptor; NFAT, nuclear factor of activated T cells; AP, activator protein; NK, natural killer; ZAP, zeta-chain-associated protein kinase; TAM, tumor-associated macrophage; PD, programmed cell death protein; CTLA, cytotoxic T-lymphocyte-associated antigen; TIGIT, T cell immunoreceptor with Ig and ITIM domains; CCL4, C-C motif chemokine ligand; IL, interleukin; Lck, lymphocyte-cell-specific protein-tyrosine kinase; BBB, blood-brain barrier; sTn, sialyl-Tn; TGF, transforming growth factor; TMED2, transmembrane p24 trafficking protein 2; KO, knockout; ZIKV, zika virus; NSCLC, non-small cell lung cancer; DAP12, dnax-activating protein of 12 kDa.

## Therapeutic Strategies

### Direct Targeting of Siglec Expression and Activity

Extensive characterization of the Siglec/sialic-acid axis’ involvement in tumor immunosuppression has motivated various attempts at targeted disruption of the Siglec receptor. For example, directly inhibiting Siglec-E, the murine counterpart of Siglec-9, with antibodies increases anti-tumor phagocytic activity, potentiates inflammatory cytokine signaling, and prolongs survival in murine GBM models and human GBM explants.^75^  *In vivo* studies in other cancer models, such as murine adenocarcinoma, have shown that antibodies targeting Siglec-7/9 can generate potent antitumor activity.^35^^,^^76-79^ Although antibodies against Siglec-7/9 have not been studied in GBM, the enrichment of Siglec-7/9 in GBM indicates that this therapy could be effective.

Targeting the CD24-Siglec-10 axis has also proven attractive given its parallels to other checkpoint pathways. TAMs *in vivo* display a more complex phenotype than the simplified M1/M2 model;^40^ in GBM, metabolic reprogramming of TAMs has emerged as a strategy. One study showed that cholesterol accumulation in TAMs upregulates Siglec-10 and PD-1, suppressing phagocytosis. An engineered oncolytic adenovirus expressing apolipoprotein A1 (ApoA1) increased GBM cell secretion of local ApoA1, restoring cholesterol efflux in TAMs, downregulating “don’t eat me” receptors, and reactivating macrophage phagocytosis through pro-inflammatory polarization, leading to durable antitumor immunity.^80^ Direct blockade of CD24-Siglec-10 binding, while uninvestigated in GBM, has been demonstrated in other cancer models; treatment with anti-CD24 antibody or CD24/Siglec-10 blocking peptide (CSBP) have both been shown to upregulate phagocytosis, enhancing engulfment of tumor cells and T cell activation.^81^^,^^82^

Earlier discussed antitumoral effects demonstrated by inhibition of Siglec-15 should motivate its investigation as a clinical target;^59^^,^^60^ Siglec-15-blocking antibodies (NC318) are already in clinical trials for NSCLC, with early evidence of clinical activity.^17^ To date, use of anti-Siglec-15 antibody in GBM has remained preclinical, with one study finding tumor regression when Siglec-15 inhibition is combined with ZIKV oncolytic therapy.^83^ Another strategy has involved modulation of tumor-suppressor microRNAs (miRNAs) that regulate Siglec-15 expression in gliomas, offering potential avenues for combination or targeted therapies.^84^

### Sialylation Modulation

Aside from targeting Siglec receptor expression, modulation of GBM’s sialylation profile has also emerged as a potential strategy. For example, inhibiting sialyltransferase activity has shown therapeutic potential. Treatment with 3Fax-Peracetyl Neu5Ac (FAX), a sialyltransferase inhibitor, selectively decreases BTIC growth.^12^ FAX has also shown the ability to disrupt GBM cell network formation, thereby limiting GBM tumor formation and reducing GBM cell migration in neocortical slice models.^13^ This highlights the potential of targeting *ST6GAL1*, as specific inhibitors are under development.^12^^,^^13^

Another approach involves targeting α2,3-sialylated glycans using specific lectins.^19^ Maackia amurensis lectin II (MAL-II) binds to α2,3-sialylated glycans (MAL-SG), which are expressed at higher levels in GSCs but drastically decrease during differentiation.^19^ Treatment of GSCs with MAL-II suppresses cell viability and sphere formation by inducing cell cycle arrest and apoptosis.^19^ Similar cytotoxic effects were observed when GSCs were treated with FAX, further suggesting that sialylation disruption can affect GSC survival and maintenance.^19^ This indicates that MAL-SG could serve as a potential biomarker and therapeutic target for suppression of GSCs, with MAL-II as a candidate anti-cancer drug.^19^ Furthermore, suppression of sialylation has also been shown to enhance the sensitivity of GBM cells to chemotherapeutic drugs like cisplatin and 5-fluorouracil.^21^

Direct administration of neuraminidase, an enzyme that cleaves terminal sialic acids, has demonstrated efficacy in preclinical models. In a GBM organotypic slice model, neuraminidase significantly reduced tumor size and the recurrence of tumor after surgical removal.^11^ It was also found to reduce ganglioside expression in GBM GSC lines, affecting cell surface glycosylation, impacting cell-cell and cell-matrix interactions, and demonstrating an overall pleiotropic action that positions it as an attractive potential treatment.^11^

Conversely, studies have also investigated the effect of inhibiting or knocking out sialidases. Inhibition of sialidases with N-acetyl-2,3-dehydro-2-deoxyneuraminic acid (DANA) leads to enhanced cellular calcium spikes and the formation of a densely connected GBM cell tumor network,^13^ implicating altered sialylation levels not only in GBM survival but also tumor formation and migration patterns.^13^ Genetic knockout of sialidases such as *NEU1* or *NEU4* in GBM cells leads to significantly higher *de novo* cell sialylation and promotes tumor growth and intracellular calcium activity as well.^13^

### Combination Immunotherapies

Mei et al. found that targeting Siglec-E enhanced PD-1 pathway blockade. Deletion of Siglec-E reprogrammed macrophages to a pro-inflammatory state, improved antigen presentation, increased secretion of CXCL9, CXCL10, CCL3, and CCL4, and raised co-stimulatory signals such as CD40, OX40L, and 4-1BBL. This environment activated CD4+ and CD8+ T cells and worked synergistically with anti-PD-1 or anti-PD-L1 to extend survival in intracranial and flank tumor models. These findings support Siglec-9 as a myeloid checkpoint that limits the benefit of blocking antibodies against PD-1 or PD-L1.^18^ In addition, Schmassmann et al. in 2023 found that Siglec-E on microglia and monocyte-derived cells suppresses phagocytosis and cross-presentation. Its deletion increased tumor uptake by myeloid cells, improved CD8+ T cell priming, and prolonged survival. This activation drove compensatory upregulation of PD-1 on T cells and PD-L1 on tumor and immune cells, creating a clear rationale for combination strategies. Pairing Siglec-E loss with anti-CD47 and anti-PD-1 achieved longer survival and ­complete, durable rejections on rechallenge, showing that ­coordinating innate checkpoints with PD-1 blockade can overcome GBM resistance.^75^

Further preclinical data show direct synergy between PD-1 blockade and Siglec-15 targeting. In a murine glioma model, anti-PD-1 therapy demonstrated greater efficacy in Siglec-15 knockout mice than in wild-type mice or in untreated Siglec-15 knockout mice. Early clinical results with the previously mentioned NC318 antibody include responses in PD-1-refractory non-small cell lung cancer, supporting the translational potential of combined checkpoint therapy.^59^ Beyond PD-1, consideration can be extended to other exhaustion markers, including TIM-3, which is frequently co-expressed with Siglec-5/7/9 on TAMs in gliomas.^37^ Potential investigational drugs for combined targeting include Fc-engineered monoclonal antibodies targeting Siglec-7 and Siglec-9, as well as clinical-stage anti-TIM-3 antibodies such as sabatolimab and TSR-022.^76^^,^^77^ Sialidase-conjugated antibodies that disrupt Siglec-ligand interactions are also in preclinical development.^77^ The co-expression and functional redundancy of Siglecs and TIM-3 in glioma TAMs support the rationale for dual blockade, and early data ­suggest that patients with high expression of both axes may derive the greatest benefit from such combination strategies.^18^^,^^37^^,^^78^

Siglec-16 expression associates with PD-L1-related transcriptional signatures rather than those related to TIM-3, suggesting a myeloid axis that complements T cell checkpoints such as CTLA-4.^37^^,^^79^^,^^82^ Disrupting Siglec-16 should diminish tolerogenic myeloid activity and, when paired with CTLA-4 blockade, amplify T cell priming and effector functions.^83^^,^^85^ Future trials could combine Siglec-16 antagonists with ­ipilimumab or tremelimumab, enriched for Siglec-16-high, mesenchymal IDH-wildtype gliomas, and equipped with ­pharmacodynamic assays of myeloid reprogramming and T cell activation, while proactively managing the intracranial immune toxicities seen with CTLA-4 inhibitors.^37^^,^^79^^,^^81^^,^^82^^,^^85^^,^^86^

Siglec inhibitors utilized in GBM studies described above are summarized in Table 2 and Figure 2. Current clinical trials investigating Siglec inhibitors for advanced tumors, albeit excepting GBM, are described in Table 3.

**Figure 2.**
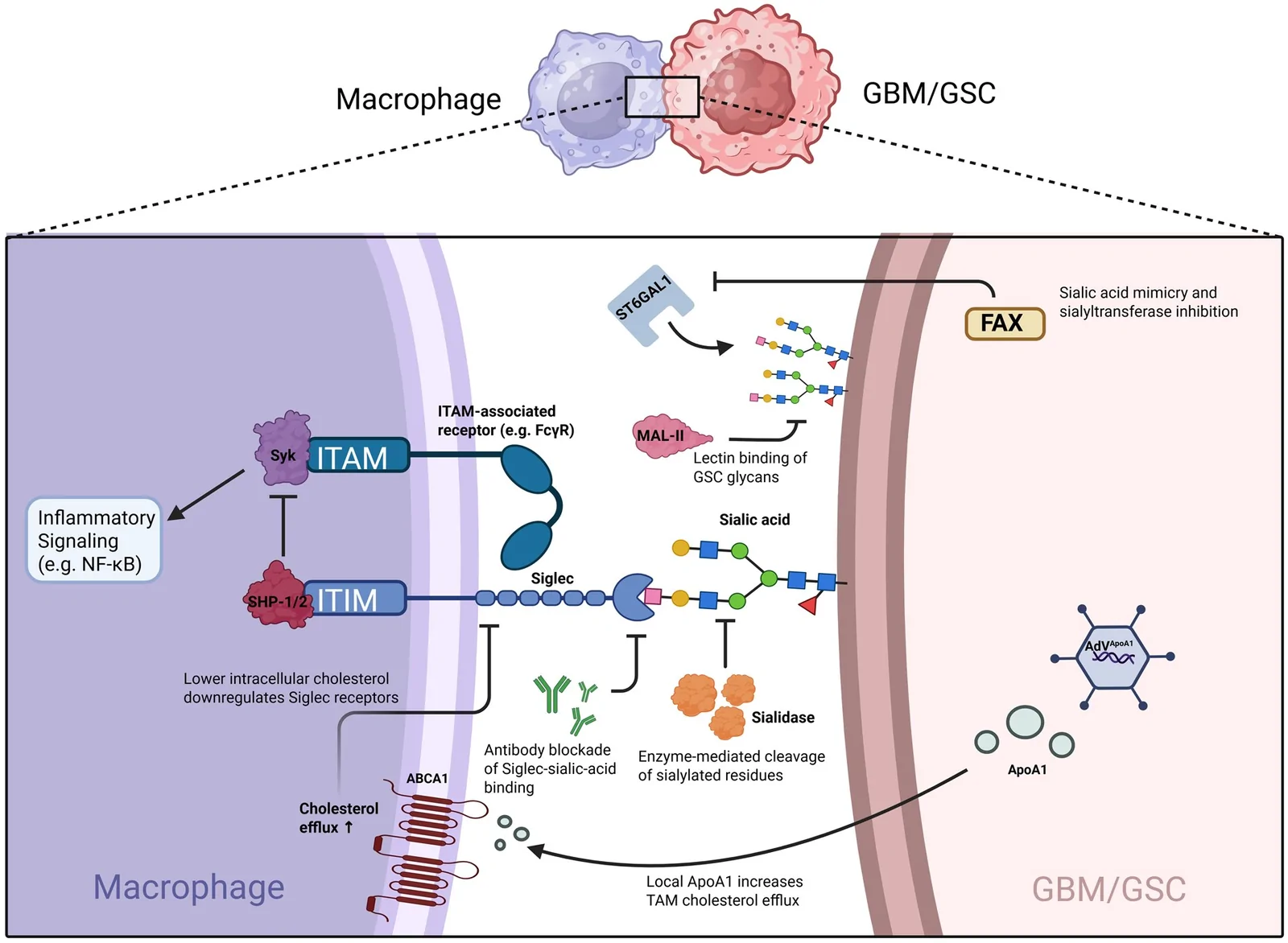
Therapies that target macrophage-anchored Siglec activity can include surface blockade, desialylation, and transcriptional modulation. Created in BioRender. Slika, H. (2026) https://BioRender.com/oimvykp. Abbreviations: GBM/GSC, glioblastoma/glioma stem cell; ST6GAL, ST6 beta-galactoside alpha-2,6-sialyltransferase; FAX, 3fax-peracetyl neu5Ac; Syk, spleen tyrosine kinase; ITAM, immunoreceptor tyrosine-based activation motif; MAL, maackia amurensis lectin, NF-κB, nuclear factor kappa-light-chain-enhancer of activated B cells; ITIM, immunoreceptor tyrosine-based inhibitory motif; SHP, src homology region 2 domain-containing phosphatase; ABCA1, ATP-binding cassette transporter A1; ApoA1, apolipoprotein A-I; TAM, tumor-associated macrophage; AdV, adenovirus.

**Table 2.** Summary of current Siglec-inhibiting drugs and platforms

Drug/platform	Molecular target	*In-vitro* GBM experiments (key details)	*In-vivo* GBM experiments & results	Combination Experiments
Anti-Siglec-9/E antibody^75^	- Siglec-9/E on TAMs	- Antibody addition to human GBM explants and cocultures increases myeloid phagocytosis, antigen presentation, and pro-inflammatory cytokines	- Antibody blockade slows tumor growth and prolongs survival; enhances cross-presentation	- Combination of Siglec-E blockade with anti-PD-1 and/or anti-CD47 yield durable tumor rejections
3Fax-Peracetyl Neu5Ac (FAX)^12^ ^,^ ^13^	- Sialyltransferases on BTICs - Functionally disrupts ST6GAL1 axis	- Reduces α2,6-sialylation on BTICs and selectively decreases BTIC growth and self-renewal vs non-BTICs - Lowers global *de novo* sialylation in GBM stem cells, slowing migration and electrical network activity	- Reduces tumor sialylation and functional network connectivity in *ex vivo* model	N/A
Neuraminidase (sialidase) enzyme^11^	- Cleaves terminal sialic acids on cancer cells to induce global desialylation	- Reduces ganglioside expression on GBM GSC lines - Disrupts cell-cell/matrix interactions via modulation of cell surface glycosylation	- Substantially reduces tumor size and regrowth potential in GBM organotype slice model	N/A
Maackia amurensis lectin II (MAL-II)^19^	Binds α2,3-sialylated glycans on GBM GSCs	- Treatment reduces viability and sphere formation of GSCs - Induces cell-cycle arrest and apoptosis	N/A	N/A
ApoA1-containing type 5 adenovirus (AdV^ApoA1^)^80^	- Infects cancer cells to produce local ApoA1 - Increased intratumoral ApoA1 levels induce cholesterol efflux in TAMs and downregulate ‘don’t eat me’ receptors	- Infection of GBM cells increases culture ApoA1 and downregulates Siglec-10 on TAMs - Improved phagocytosis by TAMs in coculture	- Intratumoral delivery reduces PD-1 expression on TAMs - Increases tumoral T-cell infiltration and activation - Increased overall antitumor immunity and prevented recurrence upon rechallenge	N/A

Abbreviations: TAM, tumor-associated macrophage; GBM, glioblastoma; PD, programmed cell death protein; BTIC, brain tumor initiating cell; ST6GAL, ST6 beta-galactoside alpha-2,6-sialyltransferase; GSC, glioma stem cell; ApoA1, apolipoprotein A1.

**Table 3.** Siglec-inhibiting drugs currently in clinical trials

Drug/platform	Molecular target	Cancer indications in trials	Trial phase/status (NCT)	Summary outcomes (publicly reported)	Combination tested?
NC318 (anti-Siglec-15 mAb, NextCure)^94^ ^,^ ^95^	Siglec-15	Advanced solid tumors with NSCLC focus	Phase 1/2 (NCT03665285); Phase 2 combo with pembrolizumab (NCT04699123)	- Early disease-control signals and activity in PD-1–refractory NSCLC reported in abstracts and company updates - Monotherapy signals are mixed, with limited efficacy noted in later analyses	- Combined with pembrolizumab (PD-1) in Phase 2.
PYX-106 (anti-Siglec-15 mAb, formerly BSI-060T, Pyxis Oncology)^96^	Siglec-15	Advanced solid tumors	Phase 1 (FIH) (NCT05718557)	- Outcomes of interest include safety, PK/PD, and biomarker work - Efficacy readouts pending	N/A
ONC-841 (anti-Siglec-10 mAb, OncoC4)^50^	Siglec-10	Advanced or metastatic solid tumors	Phase 1 (FIH) (NCT06352359)	- First patient dosed Aug 2024 - Dose-escalation safety/PK ongoing, but no data released yet.	N/A

Abbreviations: mAb, monoclonal antibody; NSCLC, non-small cell lung cancer; PD, programmed cell death protein.

### Non-Immunotherapeutic Combinations

Translational data present a rationale for pairing standard glioma drugs with Siglec-directed therapy. In glioma-microglia/monocyte transwell cultures, temozolomide (TMZ) reprograms cytokine and arginase profiles, remodels Siglec transcription and surface expression, and increases tumor binding of inhibitory Siglec-5/11 Fc probes. These changes heighten dependence on the sialoglycan-Siglec checkpoint and prime tumors for Siglec blockade.^87^^,^^88^ Independently, disrupting the Siglec-9/Siglec-E axis in murine GBM boosts phagocytosis, cross-presentation, pro-inflammatory cytokines, T cell priming, and survival, acting additively with other immunotherapies and providing a mechanistic foundation for combining Siglec inhibition with TMZ.^75^ Reviews further highlight TMZ’s dual immune profile consisting of local immunogenic cell death versus systemic lymphodepletion and myeloid skewing, which underscores the need for concurrent myeloid-checkpoint blockade.^89^

Dexamethasone (Dex) presents the opposite problem. *In vitro*, Dex amplifies α2,8-sialylation on glioma cells, up-regulates microglial Siglec-E, and strengthens Siglec-E fusion-protein binding, deepening the sialoglycan-Siglec brake and thereby creating a logical niche for Siglec blockade to restore phagocytosis and antigen presentation.^90^ Human co-culture systems corroborate such immune inversion, showing Dex-induced shifts in Siglec expression, ­cytokines, arginase phenotypes, and enhanced inhibitory Siglec-5/11 engagement.^87^ Large-scale transcriptomics ­likewise reveal Siglec-5/7/9/16 enrichment in high-grade, IDH-wild-type, mesenchymal gliomas, supporting biomarker-driven trials of these combinations.^37^

Post-operative external-beam radiotherapy (60 Gy/30 fractions or hypofractionated variants) remains the cornerstone of GBM management.^91^ Beyond cytoreduction, radiation therapy releases tumor antigens, induces type I-interferon signaling, and encourages T cell priming.^92^^,^^93^ These same events complement Siglec blockade. In GBM models, inhibiting the Siglec-sialic axis reprograms microglia and monocyte-derived cells, boosts phagocytosis and cross-presentation, and heightens downstream T cell responses.^75^ Radiation therapy supplies a surge of immunogenic debris that these reconditioned myeloid cells can more ­efficiently process, converting local tumor kill into systemic antitumor immunity. It may also transiently reshape glycans and myeloid phenotypes, exposing additional opportunities in which inhibitory Siglec signaling becomes druggable.^88^^,^^93^

## Conclusion

GBM’s interaction with the Siglec/sialic-acid axis is increasingly recognized as a driver of immune suppression and immunotherapy resistance. Simultaneous hypersialylation of the GBM TME and upregulation of inhibitory Siglecs on tumor-infiltrating immune cells (ie macrophages, microglia, MDSCs, T cells) create a complex network of redundant, immune-suppressive interactions that persist despite blockade or ablation of traditional immune checkpoints like PD-1. Preclinical evidence, increasingly involving GBM-related models, demonstrates that disruption of these interactions, either through Siglec targeting or GBM glycome modulation, can restore antitumoral macrophagic phenotypes and cytotoxic signaling. Moreover, the addition of transcriptomic and single-cell expression analyses reinforces experimental data implicating potential synergy between the Siglec/sialic-acid axis and other immunotherapies, including PD-1 or CTLA-4 blockade, as well as radiotherapy or TMZ. These findings provide sufficient justification for the pursuit of further translational studies. The novelty of this axis in oncological study is reflected in a lack of Siglec-antagonizing agents, let alone those that would be deliverable across the BBB. Moreover, mechanistic insights into the specific ligands recognized by Siglecs, such as the unidentified Siglec-15 ligand, require further elucidation. Ultimately, disruption of the Siglec/sialic-acid axis may serve as an avenue for the reprogramming of a myeloid-dominated GBM TME and broad destabilization of GBM’s immune-suppressive capabilities.

## Data Availability

No new data were generated or analyzed in support of this research.
